# Circulating immune biomarkers correlating with response in patients with metastatic renal cell carcinoma on immunotherapy

**DOI:** 10.1172/jci.insight.185963

**Published:** 2025-01-07

**Authors:** Joyce Hwang, Eda Holl, Yuan Wu, Anika Agarwal, Mark D. Starr, Marco A. Reyes Martinez, Andrew Z. Wang, Andrew J. Armstrong, Michael R. Harrison, Daniel J. George, Andrew B. Nixon, Tian Zhang

**Affiliations:** 1 Division of Medical Oncology, Department of Medicine,; 2 Department of Surgery, and; 3 Department of Biostatistics, Duke University, Durham, North Carolina, USA.; 4 The University of North Carolina System, Chapel Hill, North Carolina, USA.; 5 Duke Cancer Institute Center for Prostate and Urologic Cancers, Durham, North Carolina, USA.; 6 Division of Medical Oncology, Department of Medicine, Duke University, Durham, North Carolina, USA.; 7 Department of Radiation Oncology and; 8 Division of Hematology and Oncology, Department of Internal Medicine, University of Texas, Southwestern Medical Center, Harold C. Simmons Comprehensive Cancer Center, Dallas, Texas, USA.

**Keywords:** Oncology, Cancer immunotherapy, Cytokines, Innate immunity

## Abstract

Since multiple front-line immune checkpoint inhibitor–based (ICI-based) combinations are approved for metastatic renal cell carcinoma, biomarkers predicting for ICI responses are needed past clinical prognostication scores and transcriptome gene expression profiling. Circulating markers represent opportunities to assess baseline and dynamic changes in immune cell frequency and cytokine levels while on treatment. We conducted an exploratory prospective correlative study of 33 patients with metastatic clear cell renal cell carcinoma undergoing treatment with ICIs and correlated changes in circulating immune cell subsets and cytokines with clinical responses to treatment. Cell frequencies and cytokine levels were compared between responders and nonresponders using unpaired parametric t tests, using prespecified alpha level of significance of 0.05. Classical monocyte subsets (CD14^+^CD16^–^), as well as 7 cytokines (IL-12/23 p40, macrophage inflammatory protein-1a, macrophage inflammatory protein-1b, vascular cell adhesion molecule-1, intercellular adhesion molecule-1, IL-8, and TNF-α) were higher at baseline for responding versus nonresponding patients. Dynamic changes in thymus- and activation-regulated chemokine (TARC), placental growth factor (PlGF), and vascular endothelial growth factor (VEGF) also correlated with patients with ICI response. In summary, macrophage-activating agents were observed to be important in ICI response and may highlight the importance of the innate immune response in ICI responses.

## Abstract 2

Circulating proteins and immune cell subsets in the bloodstream may predict for responses to immune treatments in metastatic kidney cancer.

## Graphical abstract

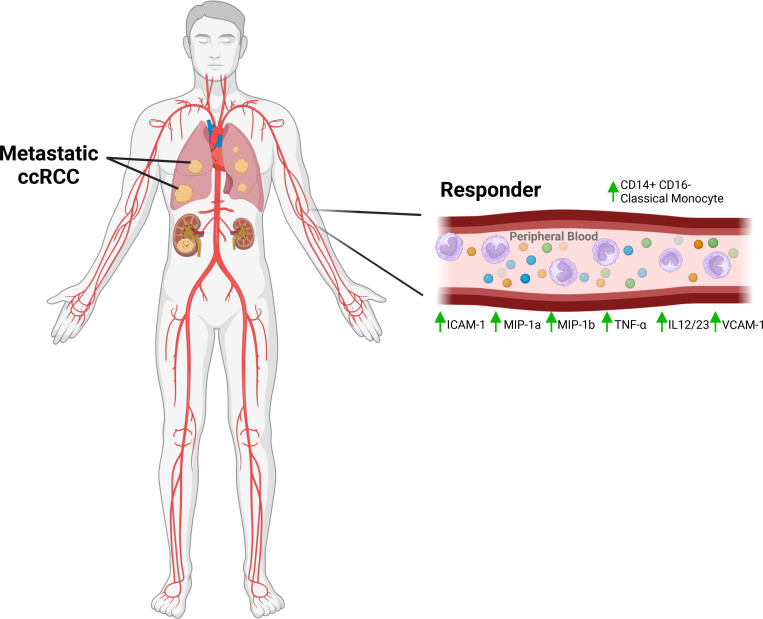

## Introduction

Renal cell carcinoma (RCC) is among the 10 most common cancers in the United States, with estimated annual cases of more than 81,800 and 14,890 deaths in 2023 (1). The landscape of therapy for metastatic RCC (mRCC) has rapidly evolved to include a number of immune checkpoint inhibitor (ICI) based regimens (2). Ipilimumab/nivolumab (ipi/nivo) (CheckMate 214), axitinib-pembrolizumab (KEYNOTE-426), axitinib-avelumab (JAVELIN Renal 101), cabozantinib-nivolumab (CheckMate 9ER), and lenvatinib-pembrolizumab (CLEAR) are all approved first-line therapies based on their overall survival benefits over VEGF tyrosine kinase inhibitors (TKIs) alone. However, durable responses to immunotherapy remain limited to a fraction of patients, and up to 75% of patients suffer severe ICI-related toxicities; therefore, biomarkers that differentiate which patients are likely to respond on ICI therapy are of great interest.

The most widely used risk-stratification model, the International Metastatic RCC Database Consortium (IMDC) criteria, were used in the trials leading to the approval of these regimens and continue to inform therapy choice. However, the IMDC criteria were defined prior to the advent of ICI therapy and do not reflect the complexity of immune host substrates or perturbations that are likely to be important for outcomes on ICI therapy. Efforts to derive markers associated with improved outcomes with ICI therapy have included transcriptomics analyses of tumors of patients in the IMmotion 150/151 trial of atezolizumab-bevacizumab versus sunitinib (3, 4), which found that atezolizumab-bevacizumab improves clinical benefit in tumors with high T effector cell and proliferation-related genes. Molecular analyses of baseline tumor samples from the JAVELIN Renal 101 trial of avelumab-axitinib versus sunitinib (5) identified a 26–gene expression signature associated with improved progression free survival on avelumab-axitinib, composed of genes related to T cell and NK cell immune responses, cell trafficking, and inflammation. However, the JAVELIN Renal 101 gene expression signature had limited overlap with the IMmotion151 T effector cell signature (5). In addition, neither the JAVELIN Renal 101 nor IMmotion 150/151 gene expression signatures predicted response in an analysis of pretreatment tumor samples from CheckMate 214 (6). Although multiple prospective phase III trials and independent studies have attempted tissue-based biomarker analyses, there are only case series of circulating immune profiling studies, including cytokine analyses, without peripheral immune cell profiling. Thus, circulating immune biomarkers that predict response to immune checkpoint therapy remain an area of unmet need.

We sought to elucidate circulating immune biomarkers in patients with mRCC on ICI therapy. Our approach was to measure baseline and changes in both circulating immune cell subsets and cytokine levels as a measure of their biologic activity at prespecified time points during ICI therapy and to correlate these findings with clinical response.

## Results

**Figure 1:**
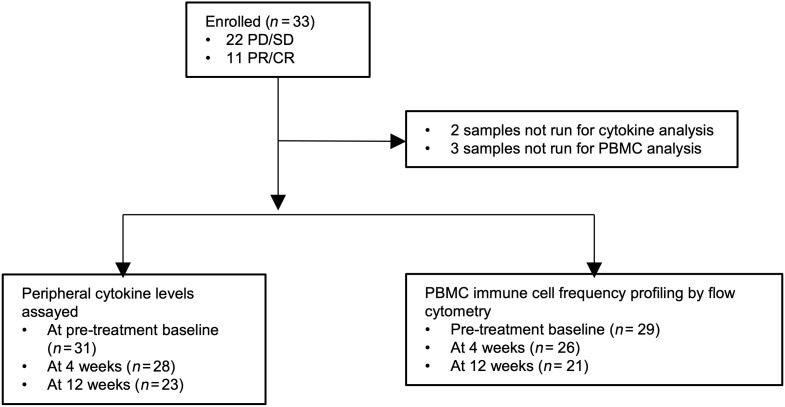
CONSORT diagram of enrollment of participants in study and sample collection.

We collected peripheral blood samples and performed flow cytometry and cytokine analysis on 33 patients with mRCC, treated with ICI-based regimens at Duke Cancer Institute clinics (Figure 1). The majority of patients were White and male, with IMDC intermediate-risk disease (Table 1). Ipi/nivo was the most common ICI-containing regimen utilized. Of the patients treated in the refractory setting, 9 patients received nivolumab and were all ICI naive, with 3 receiving prior IL-2 and 1 patient receiving ipi/nivo who was also ICI naive; the single patient who received cabozantinib/nivolumab was treated in the fourth line setting and had received prior atezolizumab as ICI. Three patients had sarcomatoid or rhabdoid features on pathology. Peripheral blood was collected at the start of treatment, at 4 weeks on treatment, and at 12 weeks on treatment.

**Figure 2:**
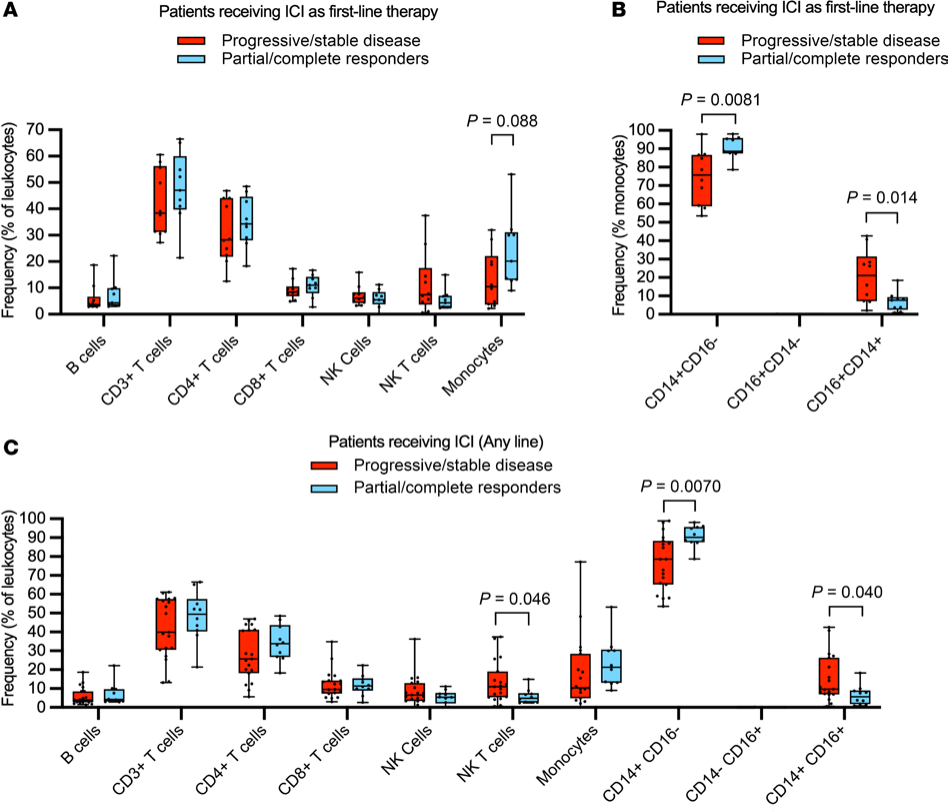
Immune cell subsets in patients with metastatic renal cell carcinoma receiving immune checkpoint inhibitor (ICI) therapy. (**A**) Frequency of circulating immune cell subsets as percentage of peripheral blood mononuclear cells in responders (blue bars, *n* = 9) versus nonresponders (red bars, *n* = 10) in patients receiving ICI as first-line treatment. (**B**) Frequency of monocyte subsets as percentage of monocytes in ICI responders (blue bars, *n* = 9) versus nonresponders (red bars, *n* = 10) in patients receiving ICI as first-line treatment. (**C**) Frequency of circulating immune cell subsets in ICI responders (blue bars, *n* = 10) versus nonresponders (red bars, *n* = 19) as percentage of peripheral blood mononuclear cells in patients receiving ICI as any-line therapy. Data are represented as box and whisker plots in which whiskers represent maximum and minimum, bounds of boxes indicate 25th and 75th quartiles, and the line within box indicates the median. Frequencies between responders and nonresponders were compared by unpaired, 2-sample, 2-tailed *t* test.

To identify immune cell subsets enriched in responders, frequencies of circulating B cells, T cells, and monocytes were compared between responders and nonresponders. Within monocytes, we additionally distinguished between classical monocyte subsets (CD14^+^CD16^–^) which are traditionally phagocytic, from nonclassical (CD14^–^CD16^+^). We categorized responders as those who achieved either a partial response or complete response (PR/CR), and the rest, comprising stable disease (SD) or progressive disease (PD) as nonresponders. We first examined the patients who received ipi/nivo, as first-line treatment did not have immune perturbations to their pretreatment baselines. Nine responders and 10 nonresponders to ipi/nivo had sufficient flow cytometry samples to compare circulating immune cells. Of patients who had received ipi/nivo as first-line treatment and had flow cytometry data available (*n* = 19), responders had a trend toward more circulating monocytes at pretreatment baseline compared with nonresponders (mean 23.8% versus 13.4% of leukocytes, *t* test *P* = 0.088, odds ratio [OR] for progression 0.53 [95% CI, 0.08–3.27]) (Figure 2A). Examination of the monocytes by subsets, specifically classical (CD14^+^CD16^–^), nonclassical (CD14^–^CD16^+^), and proinflammatory (CD14^+^CD16^+^) subsets, revealed that responders had a higher frequency of classical monocytes at baseline versus nonresponders (mean 90.6% versus 74.7% of monocytes, *t* test *P* = 0.008, OR for progression 0.21 [95% CI, 0.03–1.3]), and a lower frequency of proinflammatory monocytes versus nonresponders (mean 6.92% versus 20.8% of monocytes, *t* test *P* = 0.014, OR for progression 8.17 [95% CI, 1.17–83.38]) (Figure 2B).

Extending the analysis to include patients receiving ICI as second-, third-, or fourth-line therapy in the analysis redemonstrated a higher frequency of classical monocytes (mean 90.7% versus 76.7% of total monocytes, *t* test *P* = 0.007, OR for progression 0.04 [95% C,I 0–0.29]) and a lower frequency of proinflammatory monocytes (mean 6.42% versus 15.3% of total monocytes, *t* test *P* = 0.040, OR for progression 3.21 [95% CI, 0.67–18.74]) (Figure 2C). The expanded analysis additionally revealed a lower frequency of NK T cells in responders versus nonresponders (mean 5.8% versus 13.4% of leukocytes, *t* test *P* = 0.046, OR for progression 6.86 [95% CI, 1.28–54.84]) (Figure 2C).

**Figure 3:**
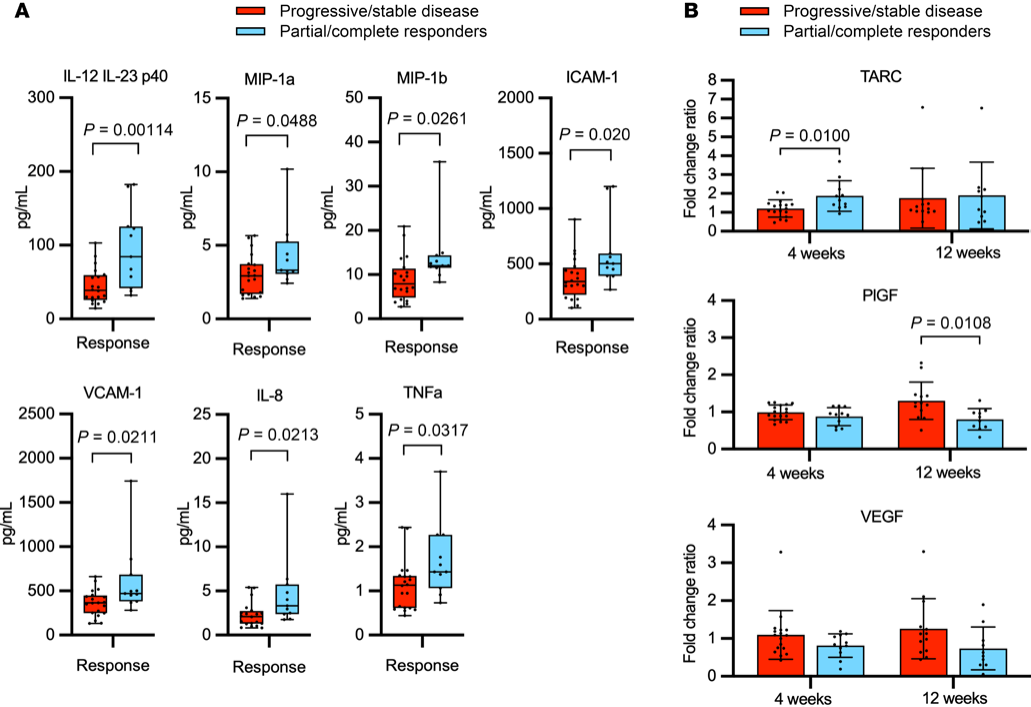
Cytokine levels that differ significantly between responders at pretreatment baseline. (**A**) Cytokine levels that differ significantly between responders (blue, *n* = 11) and nonresponders (red, *n* = 20) at pretreatment baseline. Data are represented as box and whisker plots in which whiskers represent maximum and minimum, bounds of boxes indicate 25th and 75th quartiles, and the line within box indicates the median. Frequencies between responders and nonresponders were compared by unpaired 2-sample *t* test. (**B**) Changes in thymus- and activation-regulated chemokine (TARC), placental growth factor (PlGF), and vascular endothelial growth factor (VEGF) cytokine levels while on treatment. Fold-change ratios in levels of indicated cytokine levels at 4 and 12 weeks on treatment versus pretreatment baseline, among responders (blue) and nonresponders (red). Data are shown as mean ± SD. *n* = 11 responders and *n* = 17 nonresponders at 4 weeks; *n* = 10 responders and *n* = 13 nonresponders at 12 weeks. Comparisons used unpaired parametric 2-tailed *t* tests.

To integrate our cellular findings with circulating functional proteins, we evaluated circulating cytokine levels. Seven cytokines were present at significantly higher circulating levels in responders at baseline (*n* = 11 responders, *n* = 20 nonresponders): IL-12/IL-23/p40 (mean 94.9 picograms/mL versus 44.6 pg/mL, *t* test *P* = 0.00114, OR for progression 0.2 [95% CI, 0.03–0.94]), MIP-1a (mean 4.33 pg/mL versus 3.0 pg/mL, *P* = 0.0488, OR 0.38 [95% CI, 0.08–1.69]), MIP-1b (mean 14.2 pg/mL versus 8.99 pg/mL, *t* test *P* = 0.0261, OR 0.1 [95% CI, 0.01–0.5]), VCAM-1 (mean 606 pg/mL versus 362 pg/mL, *t* test *P* = 0.0211, OR 0.2 [0.03–0.94]), ICAM-1 (mean 593 pg/mL versus 373 pg/mL, *t* test *P* = 0.020, OR 0.2 [95% CI, 0.03–0.94]), IL-8 (mean 4.70 pg/mL versus 2.30 pg/mL, *t* test *P* = 0.0213, OR 0.2 [95% CI, 0.03–0.94]), and TNF-α (mean 1.69 pg/mL versus 1.12 pg/mL, *t* test *P* = 0.0317, OR 0.2 [95% CI, 0.03–0.94]) (Figure 3A).

To identify changes in cytokine levels during treatment, fold-change ratios for each patient’s cytokine levels at 4 weeks and at 12 weeks were compared with pretreatment baseline (Figure 3B). We found that levels of thymus- and activation-regulated chemokine (TARC; CCL17) rose on treatment to a significantly greater degree in responders than nonresponders at 4 weeks (fold increase 1.87-fold versus 1.20-fold, *P* = 0.0100, *n* = 11 responders and *n* = 17 nonresponders). TARC levels also rose higher in responders than nonresponders when measured at 12 weeks (1.90-fold versus 1.75-fold), though the difference between groups at this time point did not meet statistical significance. Conversely, placental growth factor (PlGF) levels decreased in responders, whereas they rose in nonresponders such that fold-change ratios at 12 weeks were significantly different with a mean fold-change ratio of 0.799-fold in responders versus 1.30-fold in nonresponders (*P* = 0.01). Vascular endothelial growth factor (VEGF) also decreased over the course of therapy in responders and increased in nonresponders (Figure 3B), though these changes were not statistically significant.

**Figure 4:**
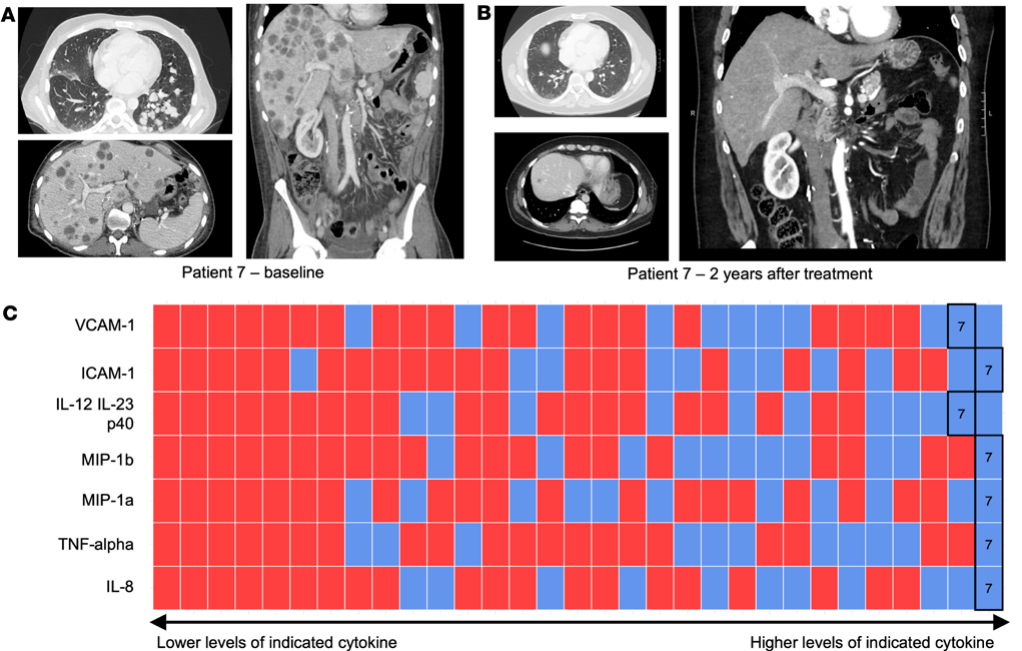
Relative cytokine levels in patient with exceptional response to ICI. (**A** and **B**) CT scan images of exceptional responder patient 7 with innumerable metastases in lungs and liver at baseline (**A**) and 2 years after treatment (**B**). (**C**) Levels of key cytokines in patients 7, 20, and 29 (3 patients with sarcomatoid/rhabdoid features) relative to those of other patients. Within each row: every patient is represented by a bar, ordered left to right by increasing level of indicated cytokine and color coded by clinical response (blue, PR/CR; red, PD/SD). Relative position of patient 7 is labeled “7”.

Our index patient, referred to here as patient 7, was noted clinically to have a particularly exceptional response to ICI therapy; at baseline, patient 7 had innumerable metastases in the lungs and liver (Figure 4A). After 2 years of treatment with ipi/nivo followed by nivolumab maintenance, the patient had resolution of all but 1 liver lesion (Figure 4B). The liver lesion also subsequently resolved, and the patient remained off treatment for 15 months without evidence of disease. Consistent with the cytokine profile identified by this work to serve as potential biomarkers of ICI response, this patient had especially high baseline levels of IL-12 IL-23 p40, macrophage inflammatory protein-1a (MIP-1a), MIP-1b, TNF-α, intercellular adhesion molecule-1 (ICAM-1), vascular cell adhesion molecule-1 (VCAM-1), and IL-8, even relative to other responders (Figure 4C). This patient had a focus of sarcomatoid dedifferentiation in his primary tumor at the time of cytoreductive nephrectomy, and indeed the 3 patients who had sarcomatoid or rhabdoid differentiation (patients 7, 20, 29) had overall higher than median levels of the 7 cytokines that were elevated in the index patient, with the exception of TNF-α and IL-8 for patient 29 (Figure 4C).

## Discussion

We conducted an exploratory analysis of peripheral immune cell frequencies and cytokine levels to identify candidate circulating biomarkers of response to ICI therapy in patients with mRCC. While all ICIs were given in patients who had not previously received immunotherapy, not all patients received ICI as first-line therapy, and there was only 1 patient who received combination ICI with a VEGF inhibitor, which has now become standard of care. A strength of our study is integrating both cellular frequencies and cytokine levels, which enhanced our sensitivity of detecting immune correlates on the basis of cell frequency or function and allowed us to assess for congruence between identified key cellular players (e.g., monocytes) and associated cytokine products or regulators. We found that patients who responded to ICIs had a higher frequency of circulating classical monocytes at pretreatment baseline compared with nonresponders. One limitation is the limited role that flow cytometry has in clinical practice for patients with solid tumors; therefore, the meaningful immune cell subsets here may not be found in routine clinical practice.

Interestingly, of the 7 cytokines that this work identified as being associated with better outcomes, 5 are produced by or activate monocytes/macrophages: IL-12 and/or IL-23 (as detected by IL-12 IL-23 p40), both MIP-1a and MIP-1b, TNF-α, and IL-8. Moreover, a patient with an exceptional response even relative to other responders (patient 7) had exceptionally high levels of all of these cytokines, and these seemed consistently elevated in patients with sarcomatoid or rhabdoid dedifferentiation. A correlation between a higher frequency of classical monocytes and better outcomes has been observed in metastatic melanoma (7); our findings support that classical monocytes are promising circulating biomarkers for predicting ICI response in RCC. To our knowledge, this is the first study to demonstrate clear differences in circulating monocytes at baseline between responders and nonresponders to ICI treatment in mRCC.

Additionally, we found that patients with fewer circulating NKT cells had better outcomes with ipi/nivo. This is interesting, given that RCC tumor expression of CD1d, the molecule that presents ligands to NKT cells, has been found to correlate with aggressive disease (8). In contrast to many other tumors (9), a lower level of circulating NK T cells in patients who have better outcomes with ipi/nivo may relate to particular aspects of RCC tumor biology.

In addition to assaying baseline cytokine levels, we asked if changes in cytokine levels over the course of treatment were associated with response. We found that responders had a greater increase in TARC (CCL17) than nonresponders after 4 weeks on therapy. Increased TARC levels have been associated with improved survival in metastatic melanoma, attributed to the role of this cytokine in recruiting T cells to the tumor sites (10, 11), our study suggests this is also true for mRCC treated with ICI. In addition, we found that PlGF decreased over the course of therapy in responders, whereas nonresponders exhibited an increase in PlGF. Of note, VEGF also decreased over the course of therapy in responders and increased in nonresponders (Figure 4), though these changes were not statistically significant. Angiogenesis inhibition and immune checkpoint inhibition are thought to act synergistically in mRCC and is part of the rationale for ICI/TKI combination therapies. The decrease in PlGF and VEGF while on ICI treatment appears to be an evaluable marker of this synergy.

Prior analyses in mRCC have similarly shown that circulating inflammatory cytokines such as IL-6 and IFN-γ, as well as circulating T cell subsets, are associated with clinical benefit (12, 13). Unlike these prior studies, our study was focused on patients treated with only immunotherapy treatments (nivolumab or ipi/nivo) and found an effect of inflammatory cytokines and immune cell subsets active in innate immunity. This current analysis was limited in the lack of interrogation on circulating tumor DNA, which has been a more recent focus of many efforts in RCC.

Finding predictive biomarkers for ICI response in mRCC and other solid tumors remains a challenge. Our study showed that both a subset of immune cells and inflammatory cytokines active in innate immunity have the potential to be critical differentiators between responders and nonresponders to ICIs. Our findings contribute to growing evidence that ICIs directly or indirectly upregulate adaptive immune responses through innate immune cells (14). With further validation in larger cohorts, these signals would improve our current prognostication and treatment selection pathways. Furthermore, these results support the importance of trials that combine treatments in activating both adaptive and innate immunity for RCC.

## Methods

### Sex as a biological variable.

Both men and women with mRCC were enrolled to this study, and similar findings are reported for both sexes.

### Study population and blood collection.

The study population consisted of patients with mRCC receiving standard-of-care immunotherapy treatment at Duke Cancer Institute. Thirty-three patients were prospectively enrolled and followed between March 2017 and March 2020. Blood was collected both prior to and during ICI treatment at baseline, 4 weeks, and 12 weeks after starting therapy. Demographic and clinicopathologic variables were recorded at the time of initial consultation (Table 1). Sample collection was performed at baseline (pretreatment), week 4, week 12, and where available, at progression of disease. Patients were subsequently categorized into PR/CR versus those categorized as nonresponders based on PD/SD at their best response using the response evaluation criteria in solid tumors (RECIST) guidelines. 

### Isolation and storage of peripheral blood mononuclear cells (PBMCs).

Blood was obtained by venipuncture and collected into CPT Mononuclear Cell Preparation vacutainers for flow cytometry analyses and EDTA vacutainers for cytokine analyses (BD Biosciences). PBMCs were isolated according to the manufacturer’s recommendations. EDTA plasma was processed within 2 hours, double-spun, aliquoted, and frozen. PBMC were viably cryopreserved and stored in vapor phase liquid nitrogen, while plasma was stored at –80°C for future use.

### Flow cytometry analysis.

Circulating immune cells were identified and quantitated using flow cytometry with DuraClone antibody panels (Beckman Coulter). For basic immune profiling, cells (1 × 10^6^) were stained with the DuraClone Basic antibody cocktail composed of CD3, CD4, CD8, CD16, CD19, CD45, and CD56 (Beckman Coulter, B53309) for 15 minutes. Cells were washed and data were then acquired on a CytoFlex flow cytometer. All collected data were then analyzed using Kaluza Software (Beckman Coulter). Data analysis was performed using Kaluza software (Beckman Coulter). The gating strategy is shown in Supplemental Figure 1 (supplemental material available online with this article; https://doi.org/10.1172/jci.insight.185963DS1). Flow cytometry measurements were performed blinded. Datasets will be provided upon request.

### Cytokine analysis.

Circulating biomarkers of inflammation/immune response were identified and quantitated using the Meso Scale Quickplex SQ 120 system from Meso Scale Diagnostic (MSD) LLC and the V-PLEX Human Biomarker 46-Plex (MesoScale Diagnostics, K15088D-1). Plasma was batch analyzed, and the following markers were assessed: IFN-γ, IL-1b, IL-2, IL-4, IL-5, IL-6, IL-7, IL-8, IL-10, IL-12, IL-12/IL-23p40, IL-13, IL-15, IL-16, IL-17A, IL-21, IL-22, IL-27, IL-31, MIP-3a, Eotaxin, Eotaxin-3, IP-10, MCP-1, MCP-4, MDC, MIP-1a, MIP-1b, TARC, TNF-α, TNF-β, GM-CSF, CRP, ICAM-1, SAA, VCAM-1, FGFb, FLT-1, PlGF, Tie-2, VEGF, VEGF-C, and VEGF-D. Multiplex panels were performed at the prespecified dilution, and each sample was tested in duplicate. IL-13, IL-1b, IL-17A, GM-CSF, IL-2, IL-4, IL-5, IL-23, and VEGF-C were excluded from downstream analyses because levels were below the limit of detection in 95% of patients. Cytokine measurements were performed blinded. Datasets will be provided on request.

### Statistics.

Data collection was performed blinded to response. Peripheral blood cell frequencies and cytokine levels at each time point (baseline, 4 weeks, and 12 weeks on treatment) were compared between responders and nonresponders. For each cell type, frequencies were compared between responders and nonresponders using unpaired parametric 2-tailed *t* tests, using a prespecified level of significance of *P* < 0.05. Similarly, for each cytokine, levels were compared between responders and nonresponders using unpaired parametric *t* tests. For each cytokine, fold ratios were calculated as follows: fold ratio at week 4 = cytokine level at week 4/cytokine level at baseline; fold ratio at week 12 = cytokine level at week 12/cytokine level at baseline. Fold ratios for each cytokine were compared between responders and nonresponders by multiple unpaired parametric *t* tests using a prespecified level of significance of *P* < 0.05. No correction for multiple testing was performed in this exploratory analysis. We conducted a logistic regression analysis to find the OR of nonresponders (PD/SD) versus responders (PR/CR) for each immune cell subset or cytokine, which was dichotomized at its median into high and low. An OR of < 1 means that patients with a high level are less likely to have disease progression. The *t* testing was performed in Prism GraphPad Version 9.5.0.

### Study approval.

Sample collection and biomarker analyses were approved by the Duke University IRB (Pro00076768), and informed consent was obtained from each patient. The study received ethics approval through the Duke IRB. All participants consented to participate.

### Data availability.

Deidentified data available upon request from corresponding author. Values for all data points in graphs are reported in the submitted Supporting Data Values file. All data and material will be made available upon request.

## Author contributions

Research design was contributed by EH, ABN, and TZ. JH, EH, AA, MDS, and MARM conducted experiments and acquired data. JH, EH, YW, AZW, AJA, MRH, DJG, ABN, and TZ analyzed data. Writing and editing of the manuscript were contributed by JH, EH, AZW, ABN, and TZ. MARM conducted experiments and acquired data. All authors approved the manuscript.

## Supplementary Material

Supplemental data

Supporting data values

## Figures and Tables

**Table 1:**
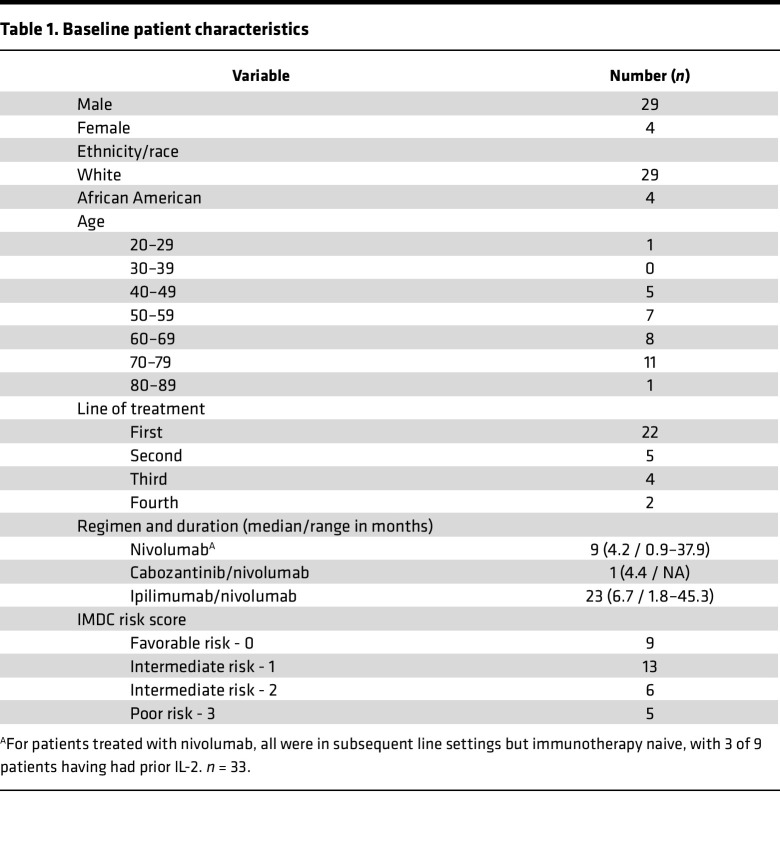
Baseline patient characteristics

## Version 1 01/07/2025

In-Press Preview

## Version 2 02/24/2025

Electronic publication
