# Supplementary material for: Circulating immune biomarkers correlating with response in patients with metastatic renal cell carcinoma on immunotherapy
Source: JCI Insight. 2025 Jan 7;10(4):e185963. doi: 10.1172/jci.insight.185963 (PMC11949027; doi:10.1172/jci.insight.185963)
Supplement: Supplemental data [file jciinsight-10-185963-s178.pdf]

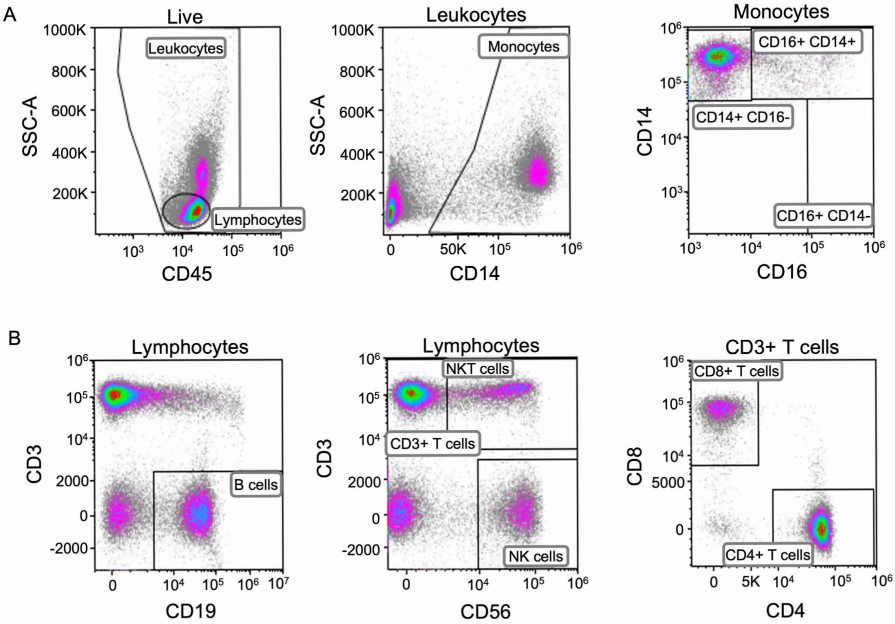

Supplemental Figure S1: Gating strategy to examine circulating immune cells in RCC patients. PBMC obtained from patients at the indicated time points were analyzed using flow cytometry. (A) Hematopoietic cells were identified via CD45 cell surface expression. Monocytes were identified and characterized based on expression of CD14 and CD16. (B) Lymphocyte subsets were identified based on CD19 (B cells), CD3, CD4 and CD8 (T cells), CD3 and CD56 (NK T cells), CD56 (NK cells).

Conflict of interest: AJA provides research support to Duke University from the NIH/NCI, PCF/Movember, DOD, Astellas, Pfizer, Bayer, Janssen, Dendreon, BMS, AstraZeneca, Merck, Forma, Celgene, Amgen, and Novartis and has consulting or advising relationships with Astellas, Epic Sciences, Pfizer, Bayer, Janssen, Dendreon, BMS, AstraZeneca, Merck, Forma, Celgene, Clovis, Exact Sciences, Myovant, and Exelixis. MRH has consulting or has advising relationships with Exelixis, AVEO, Sanofi, Telix Pharmaceuticals, Eisai, and Janssen Oncology and has received research funding from Calico, Briston-Myers Squibb, Genentech, Pfizer, Merck, Astellas Pharma, Bayer, Exelixis, Seattle Genetics, Propella Therapeutics, and Profound Bio. DJG has received research funding from Astellas, Astrazeneca, BMS, CORVUS, Exelixis, Janssen Pharmaceuticals, Novartis, Pfizer, and Surface Oncology and has consulting or advising relationships with ABRX, Astellas, Astrazeneca, Bayer H/C Pharmaceuticals, Exelixis, IdeoOncology, Janssen Pharmaceuticals, Merck Sharp & Dohme, Michael J Hennessey Associates, Propella TX, Sanofi, Seattle Genetics, Sumitovant Biopharma, and WebMD. ABN has received research funding from Genentech, Genmab, MedImmune/AstraZeneca, and Seattle Genetics and has received consultant/advisory compensation from Sanofi and Leap Therapeutics. TZ has received PI/research funding from Merck, Janssen, Astra Zeneca, Pfizer, Astellas, Eli Lilly, Tempus, ALX Oncology, Janux Therapeutics, OncoC4, and Exelixis; is on the advisory board for Merck, Exelixis, Sanofi-Aventis, Janssen, Astra Zeneca, Pfizer, Amgen, BMS, Eisai, Aveo, Eli Lilly, Bayer, Gilead, Novartis, EMD Serono, and Dendreon; and is a consultant for Pfizer, MJH Associates, Vaniam, Aptitude Health, PeerView, and Aravive.
